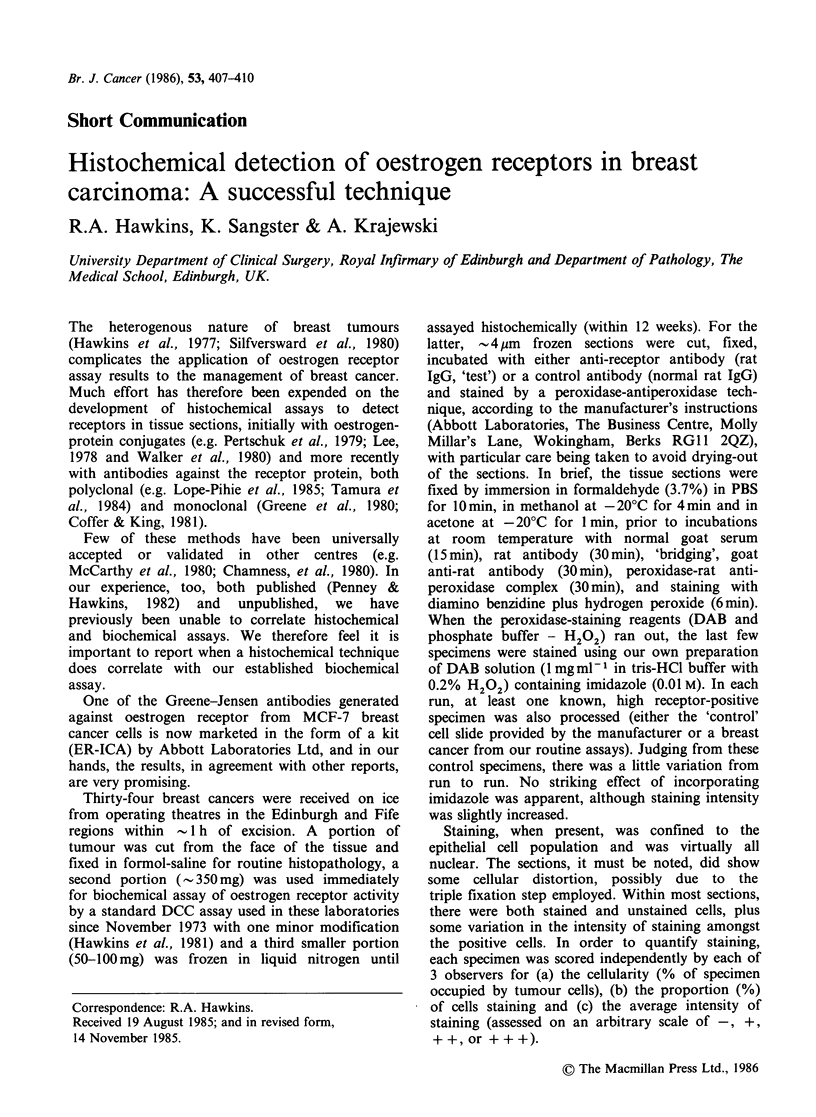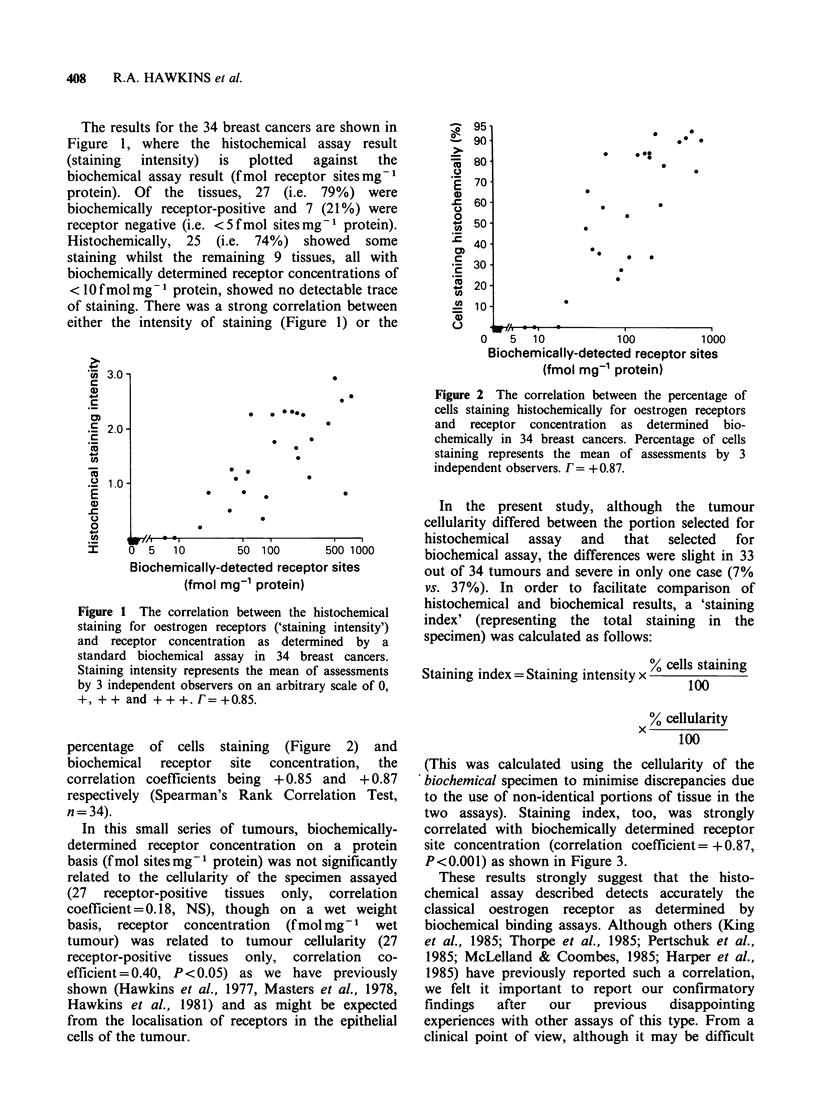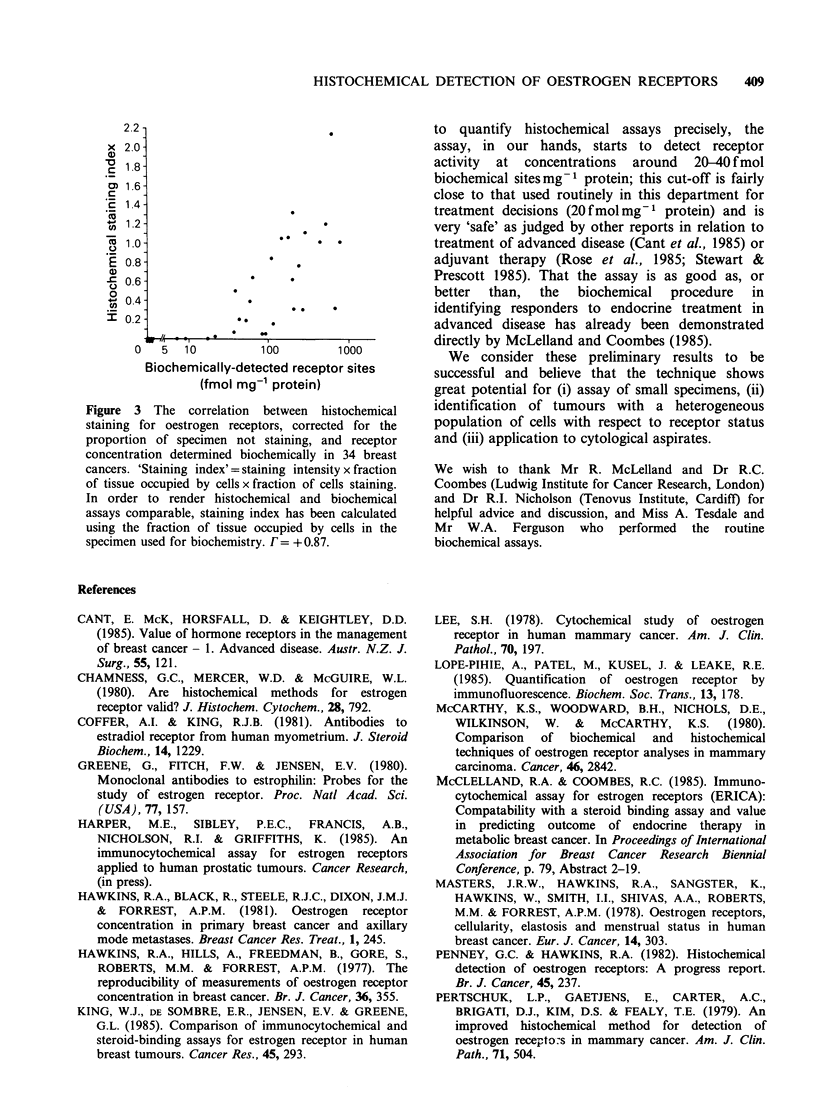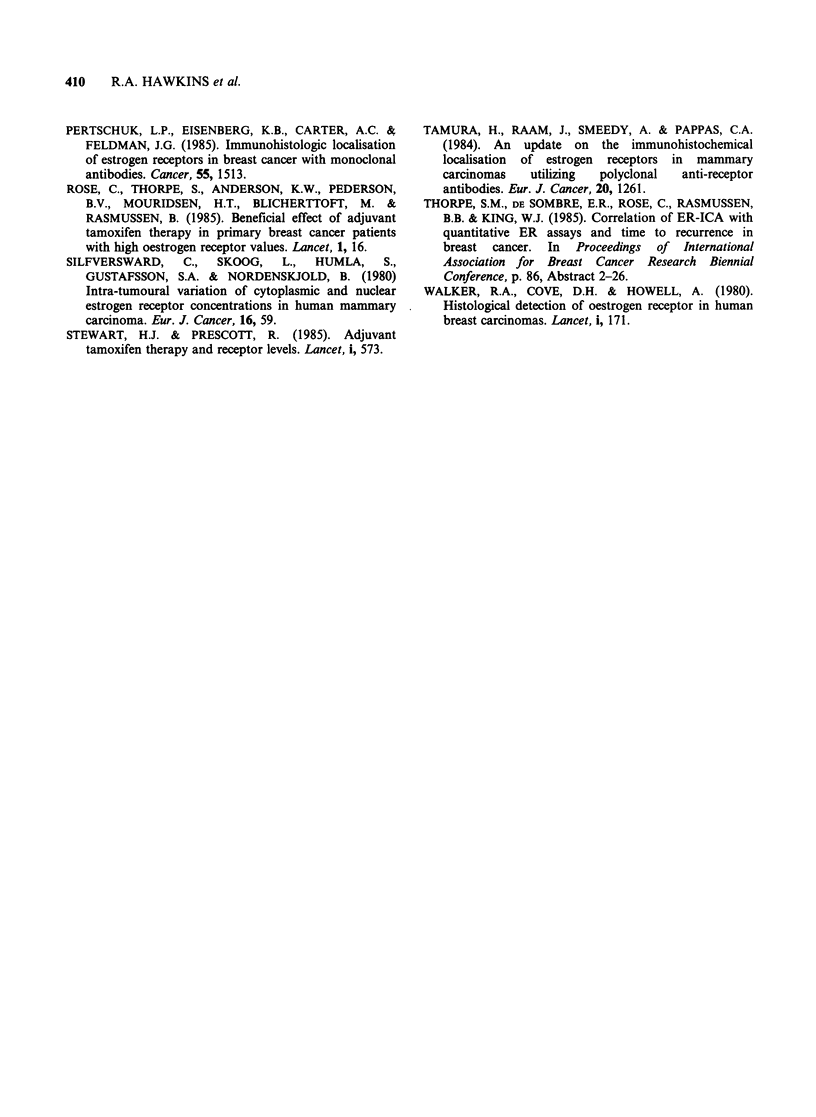# Histochemical detection of oestrogen receptors in breast carcinoma: a successful technique.

**DOI:** 10.1038/bjc.1986.66

**Published:** 1986-03

**Authors:** R. A. Hawkins, K. Sangster, A. Krajewski


*Br. J. Cancer* (1986), **53**, 407–410

### Short Communication

### Histochemical detection of oestrogen receptors in breast carcinoma: A successful technique

R.A. Hawkins, K. Sangster & A. Krajewski

*University Department of Clinical Surgery, Royal Infirmary of Edinburgh and Department of Pathology, The Medical School, Edinburgh, UK.*

The heterogenous nature of breast tumours (Hawkins *et al.*, 1977; Silfversward *et al.*, 1980) complicates the application of oestrogen receptor assay results to the management of breast cancer. Much effort has therefore been expended on the development of histochemical assays to detect receptors in tissue sections, initially with oestrogen-protein conjugates (e.g. Pertschuk *et al.*, 1979; Lee, 1978 and Walker *et al.*, 1980) and more recently with antibodies against the receptor protein, both polyclonal (e.g. Lope-Pihie *et al.*, 1985; Tamura *et al.*, 1984) and monoclonal (Greene *et al.*, 1980; Coffer & King, 1981).

Few of these methods have been universally accepted or validated in other centres (e.g. McCarthy *et al.*, 1980; Chamness, *et al.*, 1980). In our experience, too, both published (Penney & Hawkins, 1982) and unpublished, we have previously been unable to correlate histochemical and biochemical assays. We therefore feel it is important to report when a histochemical technique does correlate with our established biochemical assay.

One of the Greene–Jensen antibodies generated against oestrogen receptor from MCF-7 breast cancer cells is now marketed in the form of a kit (ER-ICA) by Abbott Laboratories Ltd, and in our hands, the results, in agreement with other reports, are very promising.

Thirty-four breast cancers were received on ice from operating theatres in the Edinburgh and Fife regions within $\sim$1 h of excision. A portion of tumour was cut from the face of the tissue and fixed in formol-saline for routine histopathology, a second portion ($\sim$350 mg) was used immediately for biochemical assay of oestrogen receptor activity by a standard DCC assay used in these laboratories since November 1973 with one minor modification (Hawkins *et al.*, 1981) and a third smaller portion (50–100 mg) was frozen in liquid nitrogen until assayed histochemically (within 12 weeks). For the latter, $\sim 4\,\mu m$ frozen sections were cut, fixed, incubated with either anti-receptor antibody (rat IgG, 'test') or a control antibody (normal rat IgG) and stained by a peroxidase-antiperoxidase technique, according to the manufacturer's instructions (Abbott Laboratories, The Business Centre, Molly Millar's Lane, Wokingham, Berks RG11 2QZ), with particular care being taken to avoid drying-out of the sections. In brief, the tissue sections were fixed by immersion in formaldehyde (3.7%) in PBS for 10 min, in methanol at $-20°C$ for 4 min and in acetone at $-20°C$ for 1 min, prior to incubations at room temperature with normal goat serum (15 min), rat antibody (30 min), 'bridging', goat anti-rat antibody (30 min), peroxidase-rat anti-peroxidase complex (30 min), and staining with diamino benzidine plus hydrogen peroxide (6 min). When the peroxidase-staining reagents (DAB and phosphate buffer – $H_2O_2$) ran out, the last few specimens were stained using our own preparation of DAB solution (1 mg ml$^{-1}$ in tris-HCl buffer with 0.2% $H_2O_2$) containing imidazole (0.01 M). In each run, at least one known, high receptor-positive specimen was also processed (either the 'control' cell slide provided by the manufacturer or a breast cancer from our routine assays). Judging from these control specimens, there was a little variation from run to run. No striking effect of incorporating imidazole was apparent, although staining intensity was slightly increased.

Staining, when present, was confined to the epithelial cell population and was virtually all nuclear. The sections, it must be noted, did show some cellular distortion, possibly due to the triple fixation step employed. Within most sections, there were both stained and unstained cells, plus some variation in the intensity of staining amongst the positive cells. In order to quantify staining, each specimen was scored independently by each of 3 observers for (a) the cellularity (% of specimen occupied by tumour cells), (b) the proportion (%) of cells staining and (c) the average intensity of staining (assessed on an arbitrary scale of −, +, + +, or + + +).

Correspondence: R.A. Hawkins.
Received 19 August 1985; and in revised form, 14 November 1985.

© The Macmillan Press Ltd., 1986

The results for the 34 breast cancers are shown in Figure 1, where the histochemical assay result (staining intensity) is plotted against the biochemical assay result (fmol receptor sites $mg^{-1}$ protein). Of the tissues, 27 (i.e. 79%) were biochemically receptor-positive and 7 (21%) were receptor negative (i.e. <5 fmol sites $mg^{-1}$ protein). Histochemically, 25 (i.e. 74%) showed some staining whilst the remaining 9 tissues, all with biochemically determined receptor concentrations of <10 fmol $mg^{-1}$ protein, showed no detectable trace of staining. There was a strong correlation between either the intensity of staining (Figure 1) or the percentage of cells staining (Figure 2) and biochemical receptor site concentration, the correlation coefficients being +0.85 and +0.87 respectively (Spearman's Rank Correlation Test, $n=34$).

**Figure 1** The correlation between the histochemical staining for oestrogen receptors ('staining intensity') and receptor concentration as determined by a standard biochemical assay in 34 breast cancers. Staining intensity represents the mean of assessments by 3 independent observers on an arbitrary scale of 0, +, ++ and +++. $r=+0.85$.

In this small series of tumours, biochemically-determined receptor concentration on a protein basis (fmol sites $mg^{-1}$ protein) was not significantly related to the cellularity of the specimen assayed (27 receptor-positive tissues only, correlation coefficient = 0.18, NS), though on a wet weight basis, receptor concentration (fmol $mg^{-1}$ wet tumour) was related to tumour cellularity (27 receptor-positive tissues only, correlation coefficient = 0.40, $P<0.05$) as we have previously shown (Hawkins *et al.*, 1977, Masters *et al.*, 1978, Hawkins *et al.*, 1981) and as might be expected from the localisation of receptors in the epithelial cells of the tumour.

**Figure 2** The correlation between the percentage of cells staining histochemically for oestrogen receptors and receptor concentration as determined biochemically in 34 breast cancers. Percentage of cells staining represents the mean of assessments by 3 independent observers. $r=+0.87$.

In the present study, although the tumour cellularity differed between the portion selected for histochemical assay and that selected for biochemical assay, the differences were slight in 33 out of 34 tumours and severe in only one case (7% *vs.* 37%). In order to facilitate comparison of histochemical and biochemical results, a 'staining index' (representing the total staining in the specimen) was calculated as follows:

$$\text{Staining index} = \text{Staining intensity} \times \frac{\%\ \text{cells staining}}{100} \times \frac{\%\ \text{cellularity}}{100}$$

(This was calculated using the cellularity of the *biochemical* specimen to minimise discrepancies due to the use of non-identical portions of tissue in the two assays). Staining index, too, was strongly correlated with biochemically determined receptor site concentration (correlation coefficient = +0.87, $P<0.001$) as shown in Figure 3.

These results strongly suggest that the histochemical assay described detects accurately the classical oestrogen receptor as determined by biochemical binding assays. Although others (King *et al.*, 1985; Thorpe *et al.*, 1985; Pertschuk *et al.*, 1985; McLelland & Coombes, 1985; Harper *et al.*, 1985) have previously reported such a correlation, we felt it important to report our confirmatory findings after our previous disappointing experiences with other assays of this type. From a clinical point of view, although it may be difficult

**Figure 3** The correlation between histochemical staining for oestrogen receptors, corrected for the proportion of specimen not staining, and receptor concentration determined biochemically in 34 breast cancers. 'Staining index' = staining intensity × fraction of tissue occupied by cells × fraction of cells staining. In order to render histochemical and biochemical assays comparable, staining index has been calculated using the fraction of tissue occupied by cells in the specimen used for biochemistry. $r = +0.87$.

to quantify histochemical assays precisely, the assay, in our hands, starts to detect receptor activity at concentrations around 20–40 fmol biochemical sites mg$^{-1}$ protein; this cut-off is fairly close to that used routinely in this department for treatment decisions (20 fmol mg$^{-1}$ protein) and is very 'safe' as judged by other reports in relation to treatment of advanced disease (Cant *et al.*, 1985) or adjuvant therapy (Rose *et al.*, 1985; Stewart & Prescott 1985). That the assay is as good as, or better than, the biochemical procedure in identifying responders to endocrine treatment in advanced disease has already been demonstrated directly by McLelland and Coombes (1985).

We consider these preliminary results to be successful and believe that the technique shows great potential for (i) assay of small specimens, (ii) identification of tumours with a heterogeneous population of cells with respect to receptor status and (iii) application to cytological aspirates.

We wish to thank Mr R. McLelland and Dr R.C. Coombes (Ludwig Institute for Cancer Research, London) and Dr R.I. Nicholson (Tenovus Institute, Cardiff) for helpful advice and discussion, and Miss A. Tesdale and Mr W.A. Ferguson who performed the routine biochemical assays.

### References

CANT, E. McK, HORSFALL, D. & KEIGHTLEY, D.D. (1985). Value of hormone receptors in the management of breast cancer – 1. Advanced disease. *Austr. N.Z. J. Surg.*, **55**, 121.

CHAMNESS, G.C., MERCER, W.D. & McGUIRE, W.L. (1980). Are histochemical methods for estrogen receptor valid? *J. Histochem. Cytochem.*, **28**, 792.

COFFER, A.I. & KING, R.J.B. (1981). Antibodies to estradiol receptor from human myometrium. *J. Steroid Biochem.*, **14**, 1229.

GREENE, G., FITCH, F.W. & JENSEN, E.V. (1980). Monoclonal antibodies to estrophilin: Probes for the study of estrogen receptor. *Proc. Natl Acad. Sci. (USA)*, **77**, 157.

HARPER, M.E., SIBLEY, P.E.C., FRANCIS, A.B., NICHOLSON, R.I. & GRIFFITHS, K. (1985). An immunocytochemical assay for estrogen receptors applied to human prostatic tumours. *Cancer Research*, (in press).

HAWKINS, R.A., BLACK, R., STEELE, R.J.C., DIXON, J.M.J. & FORREST, A.P.M. (1981). Oestrogen receptor concentration in primary breast cancer and axillary mode metastases. *Breast Cancer Res. Treat.*, **1**, 245.

HAWKINS, R.A., HILLS, A., FREEDMAN, B., GORE, S., ROBERTS, M.M. & FORREST, A.P.M. (1977). The reproducibility of measurements of oestrogen receptor concentration in breast cancer. *Br. J. Cancer*, **36**, 355.

KING, W.J., DE SOMBRE, E.R., JENSEN, E.V. & GREENE, G.L. (1985). Comparison of immunocytochemical and steroid-binding assays for estrogen receptor in human breast tumours. *Cancer Res.*, **45**, 293.

LEE, S.H. (1978). Cytochemical study of oestrogen receptor in human mammary cancer. *Am. J. Clin. Pathol.*, **70**, 197.

LOPE-PIHIE, A., PATEL, M., KUSEL, J. & LEAKE, R.E. (1985). Quantification of oestrogen receptor by immunofluorescence. *Biochem. Soc. Trans.*, **13**, 178.

McCARTHY, K.S., WOODWARD, B.H., NICHOLS, D.E., WILKINSON, W. & McCARTHY, K.S. (1980). Comparison of biochemical and histochemical techniques of oestrogen receptor analyses in mammary carcinoma. *Cancer*, **46**, 2842.

McCLELLAND, R.A. & COOMBES, R.C. (1985). Immunocytochemical assay for estrogen receptors (ERICA): Compatability with a steroid binding assay and value in predicting outcome of endocrine therapy in metabolic breast cancer. In *Proceedings of International Association for Breast Cancer Research Biennial Conference*, p. 79, Abstract 2–19.

MASTERS, J.R.W., HAWKINS, R.A., SANGSTER, K., HAWKINS, W., SMITH, I.I., SHIVAS, A.A., ROBERTS, M.M. & FORREST, A.P.M. (1978). Oestrogen receptors, cellularity, elastosis and menstrual status in human breast cancer. *Eur. J. Cancer*, **14**, 303.

PENNEY, G.C. & HAWKINS, R.A. (1982). Histochemical detection of oestrogen receptors: A progress report. *Br. J. Cancer*, **45**, 237.

PERTSCHUK, L.P., GAETJENS, E., CARTER, A.C., BRIGATI, D.J., KIM, D.S. & FEALY, T.E. (1979). An improved histochemical method for detection of oestrogen receptors in mammary cancer. *Am. J. Clin. Path.*, **71**, 504.

PERTSCHUK, L.P., EISENBERG, K.B., CARTER, A.C. & FELDMAN, J.G. (1985). Immunohistologic localisation of estrogen receptors in breast cancer with monoclonal antibodies. *Cancer*, **55**, 1513.

ROSE, C., THORPE, S., ANDERSON, K.W., PEDERSON, B.V., MOURIDSEN, H.T., BLICHERTTOFT, M. & RASMUSSEN, B. (1985). Beneficial effect of adjuvant tamoxifen therapy in primary breast cancer patients with high oestrogen receptor values. *Lancet*, **1**, 16.

SILFVERSWARD, C., SKOOG, L., HUMLA, S., GUSTAFSSON, S.A. & NORDENSKJOLD, B. (1980) Intra-tumoural variation of cytoplasmic and nuclear estrogen receptor concentrations in human mammary carcinoma. *Eur. J. Cancer*, **16**, 59.

STEWART, H.J. & PRESCOTT, R. (1985). Adjuvant tamoxifen therapy and receptor levels. *Lancet*, **i**, 573.

TAMURA, H., RAAM, J., SMEEDY, A. & PAPPAS, C.A. (1984). An update on the immunohistochemical localisation of estrogen receptors in mammary carcinomas utilizing polyclonal anti-receptor antibodies. *Eur. J. Cancer*, **20**, 1261.

THORPE, S.M., DE SOMBRE, E.R., ROSE, C., RASMUSSEN, B.B. & KING, W.J. (1985). Correlation of ER-ICA with quantitative ER assays and time to recurrence in breast cancer. In *Proceedings of International Association for Breast Cancer Research Biennial Conference*, p. 86, Abstract 2–26.

WALKER, R.A., COVE, D.H. & HOWELL, A. (1980). Histological detection of oestrogen receptor in human breast carcinomas. *Lancet*, **i**, 171.